# Promise and the Pharmacological Mechanism of Botulinum Toxin A in Chronic Prostatitis Syndrome

**DOI:** 10.3390/toxins11100586

**Published:** 2019-10-11

**Authors:** Chien-Hsu Chen, Pradeep Tyagi, Yao-Chi Chuang

**Affiliations:** 1 Department of Urology 1, Kaohsiung Chang Gung Memorial Hospital, Chang Gung University College of Medicine, Kaohsiung 83301, Taiwan; kenkochen@yahoo.com.tw; 2 Department of Urology, University of Pittsburgh School of Medicine2, Pittsburgh, PA 15213, USA; tyagpradeep@gmail.com

**Keywords:** Botulinum toxin, chronic prostatitis

## Abstract

Chronic prostatitis/chronic pelvic pain syndrome (CP/ CPPS) has a negative impact on the quality of life, and its etiology still remains unknown. Although many treatment protocols have been evaluated in CP/CPPS, the outcomes have usually been disappointing. Botulinum neurotoxin A (BoNT-A), produced from *Clostridium botulinum*, has been widely used to lower urinary tract dysfunctions such as detrusor sphincter dyssynergia, refractory overactive bladder, interstitial cystitis/bladder pain syndromes, benign prostatic hyperplasia, and CP/ CPPS in urology. Here, we review the published evidence from animal models to clinical studies for inferring the mechanism of action underlying the therapeutic efficacy of BoNT in CP/CPPS. Animal studies demonstrated that BoNT-A, a potent inhibitor of neuroexocytosis, impacts the release of sensory neurotransmitters and inflammatory mediators. This pharmacological action of BoNT-A showed promise of relieving the pain of CP/CPPS in placebo-controlled and open-label BoNT-A and has the potential to serve as an adjunct treatment for achieving better treatment outcomes in CP/CPPS patients.

## 1. Introduction

Chronic prostatitis describes a constellation of complaints such as pain in the perineum, genitalia, pelvis, or lower abdomen, ejaculation pain, or irritative/voiding urinary symptoms [1,2]. In 1995, National Institutes of Health (NIH) developed a consensus on the general definition and classification of prostatitis, chronic prostatitis/chronic pelvic pain syndrome (CP/CPPS) as the “presence of genitourinary pain in the absence of uropathogenic bacteria as detected by standard microbiologic methodology” [3]. This disease has negatively affected the quality of life in 5–10% of the adult males of North America; however, the etiology is still largely unknown [4,5].

## 2. Current Understanding of CP/CPPS Pathophysiology

Considering that CP/CPPS symptoms are not localized to a singular organ, there is mounting evidence to support that symptoms emanate from the interplay between multiple organ systems. Several possible mechanisms are postulated to cause referred pain of CP/CPPS, prominent among them is neurogenic inflammation that describes activation of prostate afferent nerves by prostate inflammation [6]. The constellation of CP/CPPS symptoms necessitates multimodal treatment approaches including α-blockers, antimuscarinics, anti-inflammatory agents, or muscle relaxants [7]. However, the failure of most approaches to effectively treat patients with chronic prostatitis is a source of great frustration. Thus, it is necessary to develop new therapy for patients with refractory CP/CPPS. The clinicians should not only pay attention to urological complaints but also evaluate if the co-existence of nonurological symptoms in the anorectal area, genital region or beyond these areas. Previous studies demonstrated the possible association between chronic pelvic pain and other chronic pain conditions such as irritable bowel syndrome. There are three proposed explanations for these conditions, including physiological dysfunction, victimization, and psychological distress [8,9]. Examining these factors perhaps can clarify the relationship between these comorbidities.

## 3. Botulinum Neurotoxin (BoNT)

BoNT is a potent toxin isolated from *Clostridium botulinum*, which can cleave SNAP-25 and block the release of some neurotransmitters (e.g., acetylcholine) from pre-junctional nerves at the neuromuscular junction. This toxin has been widely used to treat muscle dysfunction, such as strabismus [10]. In urology, more and more studies have focused on the application of BoNTs in detrusor sphincter dyssynergia (DSD) [11,12], overactive bladder (OAB) [13,14], interstitial cystitis/bladder pain syndromes [15,16], benign prostatic hyperplasia (BPH) [17,18,19,20], and CP/ CPPS [14,16,21] over the past decade. Growing evidence suggests that inhibition of exocytotic machinery by BoNT-A could modulate sensory processing, inflammation and glandular function [22,23,24,25,26]. To date, BoNT-A have been listed as a third-line treatment for refractory OAB [27] and fourth-line for interstitial cystitis/bladder pain syndromes [28] in the American Urology Association (AUA) guidelines. However, the application of BoNT-A for patients with prostate disorders is still under off-label use. In this article, we reviewed published literature documenting mechanisms of action, basic research and the clinical use of botulinum toxin in CP/ CPPS from Pubmed and used botulinum toxin, chronic prostatitis, and chronic pelvic pain as search terms. In addition to published studies, we also gathered some abstracts presented at international meetings.

## 4. Mechanisms of Botulinum Neurotoxins (BoNTs)

BoNT is produced by *Clostridium botulinum*, a Gram-positive anaerobic bacterium. BoNT includes seven kinds of antigenically distinct neurotoxins (A~G) [29,30]. Among them, BoNT-A is the most commonly used serotype in clinics with the most durable effect [13].

BoNT-A is synthesized as an inactive form of 1285 amino acids and becomes activated when it is cleaved into a light chain (50-kDa) and a heavy chain (100-kDa). Unique binding of BoNT to nerve terminals occurs due to their ability to interact with two independent receptors of the presynaptic membrane: a polysialoganglioside (PSG) and a glycosylated luminal domain of a synaptic vesicle protein that mediates BoNT internalization [30]. The mechanism of denervation by BoNT is divided into five steps: 1) binding to nerve terminals, 2) internalization within an endocytic compartment, 3) low pH-driven translocation of the L chain across the vesicle membrane, 4) release of the L chain in the cytosol by reduction of the interchain disulfide bond, and 5) cleavage of SNAREs for ensuing the blockade of neurotransmitter release and consequent neuroparalysis [29,30,31].

## 5. The Rationale for BoNT-A Application in Chronic Prostatitis

BoNT-A causes muscle relaxation by blocking the release of acetylcholine.

BoNT-A has been well known to have paralyzing effects via the blockage of acetylcholine release at the presynaptic cholinergic neuromuscular junction. The inhibitory effects of BoNT-A on both somatic and autonomic nerves have been used to treat a variety of conditions associated with muscularhypercontractility. Intramuscular injection of BoNT-A could lead to temporary chemodenervation and muscle relaxation in both striated and smooth muscles [29,30]. In 1988, Dykstra et al. first treated 11 patients with DSD due to spinal cord injury (SCI) by using urethral sphincter injection of BoNT-A [11]. In a prospective study using 100 U of BoNT-A injection into the urethral sphincter to treat patients with spinal cord lesions and DSD, decreased voiding detrusor pressure and increased maximum flow rate were observed [12]; 60.6% of patients felt satisfied with the outcomes but post-injection urinary incontinence was another concern.

Huang et al. conducted a prospective multicentre trial with a total of 59 SCI patients having both detrusor overactivity (DO) and DSD. All the patients simultaneously received intravesical (200 U) and urethral sphincter (100 U) injections of BoNT-A [32]. This trial demonstrated significant reductions in maximum detrusor pressure, urinary incontinence episode, and improvement in voiding volume after treatment. All patients could achieve complete dryness at the follow-up point of 2 weeks. In view of the effect of BoNT-A on smooth muscle relaxation, the therapeutic response in DO has been widely studied. In 2000, Schurch et al. first reported the injection of BoNT-A (200 to 300 U) into detrusor muscle to treat patients with neurogenic DO [33]. At the 6-week follow up, 17 of 19 (89%) patients achieved complete continence. Significantly increased maximum bladder capacity and decreased maximum detrusor voiding pressure were observed after injection. To date, intravesical BoNT-A injection has become the preferred option for refractory OAB and neurogenic DO.

**Figure 1:**
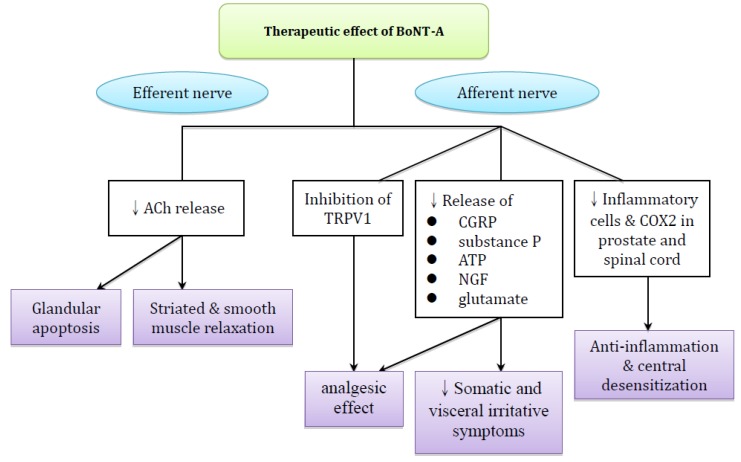
Conceivable mechanisms of BoNT-A in the treatment of CP/CPPS.

In addition to the role in blocking motor neurons, BoNT-A also has effects on the modulation of sensory nerves and inflammation as evinced by animal studies (Figure 1). A large body of evidence now supports that BoNT-A achieves analgesic effect by hindering the release of mediators responsible for painful sensation, including nerve growth factor (NGF), substance P, calcitonin gene-related peptide (CGRP), glutamate, as well as adenosine triphosphate (ATP) involved in afferent neurotransmission [22,23]. Therefore, it is postulated that BoNT-A may relieve the pain associated with chronic prostatitis by inhibiting the abnormal nociceptive neurotransmission conveyed by prostatic afferent nerves. Sensory dysfunction may be one of the postulated causes driving the symptoms of chronic prostatitis [6].

Transient Receptor Potential Vanilloid 1 (TRPV1), a non-selective cation channel, is expressed in some primary afferent neurons [34], especially in small and medium diameters (e.g., C-fibers) that are responsible for neurogenic pain and inflammation development [35]. In humans, TRPV1 activation might result in a burning pain sensation in the lower urinary tract [36]. Dinis et al. have demonstrated that abundant TRPV1 innervation are located on the prostatic urethral mucosa, verumontanum, and ejaculatory ducts [37]. Using rats in an adjuvant-arthritis pain model, Fan et al. reported that BoNT-A exerts its antinociceptive effect by reducing TRPV1 protein expression via the inhibition of plasma membrane trafficking [38]. Taken together, BoNT-A might play an important role in inhibiting TRPV1 expression in human prostate, which provides an alternative therapeutic strategy for CP/CPPS.

Regarding the anti-inflammatory effect, Chuang et al. reported that BoNT-A significantly decreased painful behavior, inflammatory cell accumulation, and cyclooxygenase (COX)-2 expression in the prostate and L6 spinal cord in dose-dependent fashion in a rat model of capsaicin- induced prostatitis [24,25]. This finding demonstrates the potential of BoNT-A in the treatment of prostate inflammation.

## 6. Clinical Studies in BoNT-A for CP/CPPS Treatment

Table 1 summarized the clinical studies of BoNT-A intraprostatic injection for chronic prostatitis. Maria el al. first reported the transperineal injection of 30 U BoNT-A into prostatic apex to treat the voiding problem in patients with chronic prostatitis in 1998 [39]. In their series, three of four patients obtained improvement in voiding without urinary incontinence with a mean follow-up duration of 12 months.

Park et al. reported intraprostatic BoNT-A injection in 84 patients with CPPS using transrectal (40 U, *n* = 78) or transperineal (200 U, *n* = 6) route [40]. Symptom improvement was found in 59% of patients with transrectal injection and in 50% with transperineal route. By the National Institutes of Health Chronic Prostatitis Symptom Index (NIH-CPSI) questionnaire, the most significant improvement was in the pain domain, followed by ejaculation-related pain. The therapeutic effect was sustained for 6~18 months. The NIH-CPSI is a 13-item questionnaire developed to assess symptoms and quality of life (QoL) in men with CP/CPPS. This questionnaire includes three domains evaluating pain, urinary complaints, and QoL.

Gottsch el al. conducted a randomized placebo-controlled study to evaluate the effect of BoNT-A (100 U) injection into perineal body and bulbospongiosus muscle for CPPS treatment [41]. One month after treatment, the response rate assessed by Global Response Assessment (GRA) was significantly better in the BoNT-A group than the placebo group (30% vs. 13%, *p* = 0.0002). Although the NIH-CPSI pain subdomain was also significantly improved in the BoNT-A group, the total NIH-CPSI score did not improve.

In a prospective, randomized, double-blind, placebo-controlled trial on transurethral intraprostatic injection of BoNT-A (100 U) for men with chronic prostatitis, Falahatkar et al. demonstrated that BoNT-A treatment group significantly improved the NIH-CPSI total and subscale scores, the American Urological Association-symptom score (AUA-SS), visual analogue scale (VAS), quality of life, and frequencies of diurnal and nocturnal urinations when compared to baseline. The most noticeable improvement was the NIH-CPSI pain subdomain and the VAS scores, which decreased by 79.9% and 82.1% at 6-month follow-up, respectively [42].

Recently, another prospective controlled study using transurethral intraprostatic injection of BoNT-A (200 U) in 43 patients also showed encouraging response rates for refractory nonbacterial CP/CPPS [43]. However, the therapeutic effect gradually declined at 9–12 months.

**Figure 2:**
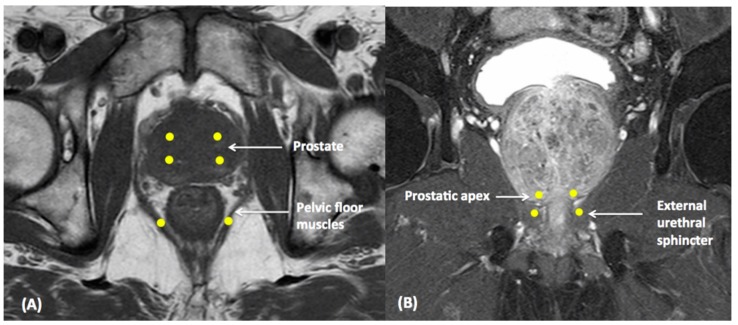
The potential injection sites of BoNT-A for CP/CPPS treatment: intraprostatic, prostatic apex, external urethral sphincter, and pelvic floor muscles. (**A**) MRI, axial view (**B**) MRI, coronal view.

As for the optimal injection route into the prostate, including transurethral, transperineal, and transrectal approaches, there is no definite suggestion so far. El-Enen et al. compared the transurethral route with transrectal intraprostatic BoNT-A injection for refractory CP/CPPS [44]. More significant improvement was observed in the transrectal group during the follow-up points. Their data also showed better results for BoNT-A injection in men with small prostates. Controversially, the aforementioned study showed that activation of TRPV1 might be one of the possible etiologies in CP/CPPS [36]. TRPV1-immunoreactive nerve fibers are distributed throughout the prostatic urethra mucosa, verumontanum, ejaculatory duct, and periurethral prostatic acini [37]. It is plausible that instead of transrectal injection, transurethral BoNT-A injection may be able to target TRPV1-immunoreactive nerve fibers as not only TRPV1-immunoreactive nerve fibers but other nerve fibers distributed in the transitional and peripheral zones may affect the efficacy of different injection routes. In addition, the drug extravasation out of the target site after injection is also an interesting issue to be discussed [45]. Further randomized and large-scale comparative studies are needed to evaluate the efficacy of different injection routes. The potential injection sites of BoNT-A for CP/CPPS treatment are shown in Figure 2.

BoNT-A injection targeting at prostate and surrounding tissue is a safe procedure when administered by an experienced injector. Side effects are always transient, like local pain, hematuria, and urinary tract infection, and in the majority of cases they are mild and tolerable [17,19,20].

## 7. Potential Impact of BoNT-A Injection on: The UPOINT Phenotype System

Clinically, it is challenging to achieve satisfactory outcomes for CP/CPPS treatment. In most cases, monotherapy has failed to successfully treat CP/CPPS, largely because of the heterogeneous nature due to multiple factors involved in the pathogenesis of CP/CPPS, such as pelvic floor muscle dysfunction, chemical irritants, as well as neurological and immunological factors. With these unsatisfactory treatment responses, combination therapies and individual consideration provide another way to improve the efficacy of treating patients with CP/CPPS. In 2009, Shoskes et al. developed the UPOINT system to classify patients with CP/CPPS to help clinicians understand the etiology and more importantly, to guide the treatment [46]. The UPOINT phenotype system includes six domains to evaluate and manage CP/CPPS: **U**rinary symptoms, **P**sychosocial dysfunction, **O**rgan-specific findings, **I**nfection, **N**eurologic/systemic complaints, and **T**enderness. Each domain has been related to specific mechanisms and treatments [7]. Patients with “urinary” complaints (storage, voiding symptoms or much post-void residual urine) can be treated with α-blocker, diet modification, and/or antimuscarinics. Intraprostatic BoNT-A can also relieve urinary symptoms [17,19,20]. “Psychosocial” patients with depression or catastrophizing evidence can be managed with counseling, cognitive behavioral therapy, antidepressants, or stress reduction. In the “organ-specific” group presenting with tenderness on prostate, increased leukocytes in prostatic fluid, blood in semen, or prostatic calcifications, some anti-inflammatory agents such as quercetin can be considered. BoNT-A has been shown to have effects on the inhibition of COX-2 expression in a preclinical prostatitis models [24,25], from which beneficial effects might reduce prostate inflammation in human CP/CPPS. Patients with “infection” (exclusion of NIH category I or II prostatitis) can be treated with antibiotics. “Neurologic/systemic” patients usually present with pain beyond abdomen and pelvis or other complaints, such as irritable bowel syndrome, fibromyalgia or chronic fatigue syndrome. In these kinds of patients, neuroleptic medications can be used to relieve symptoms. In the domain of “tenderness”, trigger points may be noted in the abdomen or pelvic floor, and treatments may include muscle relaxants use, pelvic floor physical therapy (PFPT), low-intensity shock wave, or PFPT in combination with adjuvant trigger point injection [47,48]. BoNT-A injection targeting at the pelvic muscle or trigger point might ameliorate the symptom of tenderness. Using the UPOINT system in classifying patients with CP/CPPS can facilitate hypothesis testing for possible etiologies and treatment. Shoskes et al. found that the number of positive domains was associated with severity and duration of complaints [46]. Their group also conducted a prospective study using UPOINT approach in treating 100 patients with CPPS. Eighty-four percent of patients had at least a 6-point improvement in NIH-CPSI at a median follow-up of 50 weeks [49]. Similarly, another prospective study of 140 Chinese men with CP/CPPS based on the UPOINT approach also showed a 75% response rate with a follow-up of 6 months [50]. Taken together, increasing evidence has shown that the UPOINT phenotype system is feasible and effective to clinical physicians in treating CP/CPPS. The BoNT-A prostate and pelvic muscle injection might have direct or indirect effects on the domains of **U**rinary symptoms, **P**sychosocial dysfunction, **O**rgan-specific findings, **N**eurologic/systemic complaints, and **T**enderness.

## 8. Conclusions

From basic research, it is reasonable to treat CP/CPPS with intraprostatic BoNT-A injection, based on its effects on sensory neurotransmitters and inflammation. Clinical studies have shown that results of BoNT-A treatment for CP/CPPS are promising. The most prominent improvement was on the pain subdomain of NIH-CPSI. However, the evidence is still limited and the application of BoNT-A for CP/CPPS is off-label use. Further larger randomized and placebo-controlled studies with longer follow-up periods are necessary to draw a solid conclusion.

## Figures and Tables

**Table 1:** Clinical studies of BoNT-A intraprostatic injection for chronic prostatitis.

	Maria et al. [39]	Park et al. [40]	Gottsch el al. [41]	Falahatkar et al. [42]	Abdel-Meguid et al. [43]	El-Enen et al. [44]
No. pts	4	84	29	60	43	63
Study design	prospective	prospective	randomized placebo-controlled	prospective, randomized, double-blind	Prospective two-group controlled	uncontrolled random-ised
Duration of followup (mo)	12	—	1	6	12	12
Patient criteria	spastic external urethral sphincter with poor respond to α-blocker for more than 4 months	CPPS, category IIIB	CPPS	NIH-CPSI scores ≥10 and pain subscores ≥8, refractory to 4–6 weeks’ medical therapy	refractory CP/CPPS	CP/CPPS, aged <50years, symptom duration of >2 years
BONT dose (U)	30 U	transrectal (40 U) or transperineal (200 U)	100U	prostate volumes <30 mL (100 U), 30–60 mL (200 U)	200U	100U
Injection route	transperineal	transrectal (78), transperineal (6)	transperineal	transurethral	transurethral	transurethral (28), transrectal (35)
Needle gauge	26	—	—	23	—	22
Outcomes	decrease in times of urinary flow and maximum urinary flow	NIH-CPSI improvement: transrectal (59%) and transperineal (50%). Durations of effectiveness: 6 to 18 months	Global Response Assessment (GRA): 30% response rate at 1-month; Only CPSI pain subscore reached significant improvement compared with controls	NIH-CPSI pain subdomain and the VAS scores decreased by 79.9% and 82.1% at 6-month follow-up, respectively	≥ 6 points reductions of total score of NIH-CPSI were 72.1% and 37.2% at 3 and 12 mo, respectively	good response in small prostate, short symptom duration, or transrectal route

CP/CPPS, Chronic prostatitis/chronic pelvic pain syndrome; NIH-CPSI, National Institutes of Health Chronic Prostatitis Symptom Index; AUA-SS, American Urological Association-symptom score; VAS, visual analogue scale; QoL, quality of life; “—“ indicates “not available”
